# Microdroplet-Enabled Highly Parallel Co-Cultivation of Microbial Communities

**DOI:** 10.1371/journal.pone.0017019

**Published:** 2011-02-25

**Authors:** Jihyang Park, Alissa Kerner, Mark A. Burns, Xiaoxia Nina Lin

**Affiliations:** 1 Department of Chemical Engineering, University of Michigan, Ann Arbor, Michigan, United States of America; 2 Department of Biomedical Engineering, University of Michigan, Ann Arbor, Michigan, United States of America; 3 Center for Computational Medicine and Bioinformatics, University of Michigan, Ann Arbor, Michigan, United States of America; Cinvestav, Mexico

## Abstract

Microbial interactions in natural microbiota are, in many cases, crucial for the sustenance of the communities, but the precise nature of these interactions remain largely unknown because of the inherent complexity and difficulties in laboratory cultivation. Conventional pure culture-oriented cultivation does not account for these interactions mediated by small molecules, which severely limits its utility in cultivating and studying “unculturable” microorganisms from synergistic communities. In this study, we developed a simple microfluidic device for highly parallel co-cultivation of symbiotic microbial communities and demonstrated its effectiveness in discovering synergistic interactions among microbes. Using aqueous micro-droplets dispersed in a continuous oil phase, the device could readily encapsulate and co-cultivate subsets of a community. A large number of droplets, up to ∼1,400 in a 10 mm×5 mm chamber, were generated with a frequency of 500 droplets/sec. A synthetic model system consisting of cross-feeding *E. coli* mutants was used to mimic compositions of symbionts and other microbes in natural microbial communities. Our device was able to detect a pair-wise symbiotic relationship when one partner accounted for as low as 1% of the total population or each symbiont was about 3% of the artificial community.

## Introduction

In nature, most microbes live in synergistic communities as a way to adapt to and thrive in various environments, such as the ocean[1], [2], soil[3], [4], and higher organisms as hosts[5], [6]. These microbial communities play important roles in a wide spectrum of ecosystems and form diverse interactions among community members and with their surroundings[7]. For example, the human body is a representative host for natural microbial communities: over 100 trillion bacteria are estimated to be present in the human gut[8], more than 600 microbial species are known to inhabit the human oral cavity[9], and over 100 different bacterial 16S rRNA are present on human skin[10]. These microbes are believed to be closely related to human health[11]. For instance, the gut microbiota is known to contribute to digestion of nutrients[12], stimulation of immunity[13] and protection of the host from inflammatory diseases[14]. Despite their ubiquitousness and apparent significance, our understanding of these microbial communities remains very limited, largely owing to their inherent complexity and the difficulty in laboratory cultivation of most of the microbes.

The majority of existing microbial species, estimated to be in the millions[15], have not been cultured in the laboratory[16], which severely limits the extent to which they can be characterized and further studied. One important reason behind this “unculturability” is that conventional laboratory cultivation is aimed at pure cultures of individual species, while in nature, the survival and growth of microorganisms are largely associated with their interactions with other members of the community they live in[7], [16], [17], [18]. These interactions are mediated by various molecules such as secondary metabolites, quorum sensing molecules, and peptides[19], [20], [21]. Accordingly, researchers have attempted to develop alternative cultivation techniques that allow interactions among microbes[16], [22], [23], [24], [25]. For example, Kaeberlein *et al.* successfully isolated and cultured previously uncultivated marine microorganisms by using a multi-chamber set-up which allowed the diffusion of small molecules through membranes[16].

Recent years have seen the increasing application of microfluidics, a powerful technological platform featuring small-scale and rapid operations, to cell cultivation and subsequent analysis. In particular, microfluidic compartmentalization has been widely utilized. For example, microwells have been used to confine and culture various microorganisms[26], [27], including bacteria of which the growth was quorum-sensing dependent[28]. Microfluidically generated droplets represent another strategy for creating localized environments for diverse applications such as cell cultivation[29], [30], [31], [32], screening[33] and sorting[34]. It should be noted that microbial communities have started being examined using microfluidic approaches[30]. Nevertheless, previous studies have focused exclusively on obtaining and analyzing pure cultures, which did not address the key question of how microbial interactions enable the sustenance of communities.

In this work, we aimed to make use of highly parallel micro-droplets to co-cultivate symbiotic microbial communities. We fabricated a microfluidic device that could readily encapsulate and co-cultivate subsets of a community, using aqueous droplets dispersed in a continuous oil phase. To demonstrate the effectiveness of this approach in discovering synergistic interactions among microbes, we tested it with a synthetic model system consisting of cross-feeding *E. coli* mutants, which can be used to mimic various compositions of natural microbial communities.

## Results

### Encapsulation of co-cultures in microfluidic droplets

**Figure 1:**
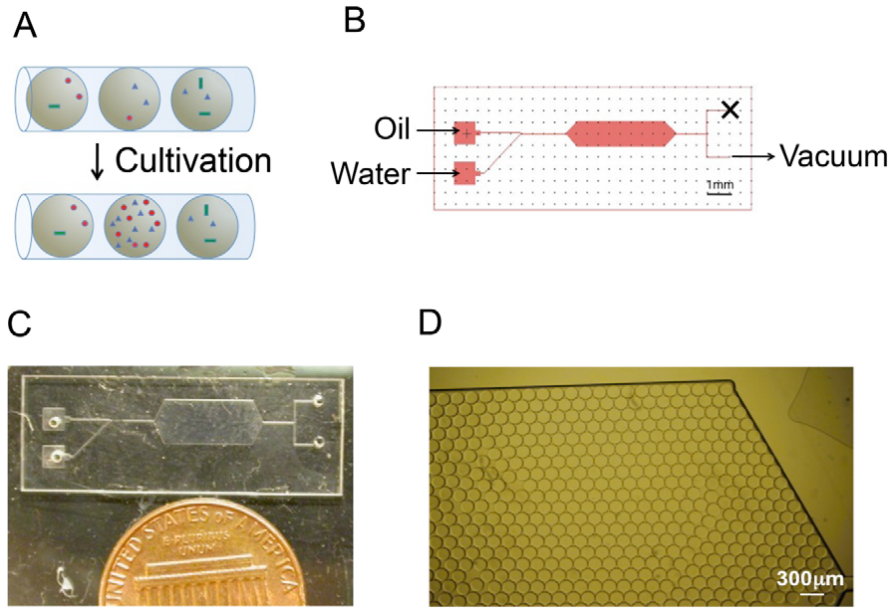
A microfluidic device for microbial co-cultivation. (A) Compartmentalized co-cultures enable detection of symbiotic relations among community members. (B) Schematic design of the microfluidic device. (C) A picture of the microfluidic device. (D) Droplets filling a large chamber in the microfluidic device.

Identification of symbiotic interactions among members in a microbial community can be facilitated by compartmentalizing and localizing the community for co-cultivation. In this work, microfluidic devices were fabricated for encapsulating and co-cultivating subsets of a microbial community in monodispersed droplets. Encapsulated microbes can grow only if the droplet localizes symbiotic interactions in it (Fig. 1a). The device comprised a slanted T-junction for droplet generation and a chamber to hold droplets for cultivation (Fig. 1b,c). The slanted T-junction is able to generate monodispersed droplets with a single vacuum line at the outlet instead of multiple lines of pressure at the inlets. Increasing power of the vacuum led to increase of the frequency of droplet generation and subsequently the number of droplets in the chamber. The achievable range of frequency was 1–500 droplets/sec. When the frequency exceeded the maximum, the droplets were no longer mono-dispersed and co-flow of two immiscible phases occurred. The volume of droplets was largely determined by the channel geometry and was maintained at about 1 nl in this work.

Droplets generated from the T-junction were collected and held in the chamber for on-chip cultivation. Surfactant was dissolved in the oil phase[35] and effectively stabilized the droplet surface. Up to ∼1,400 droplets could be packed very tightly in a 10 mm×5 mm chamber (Fig. 1d). After the droplets filled the chamber, the vacuum was removed and the flow stopped immediately. Droplets could be stably incubated for 4 days.

**Figure 2:**
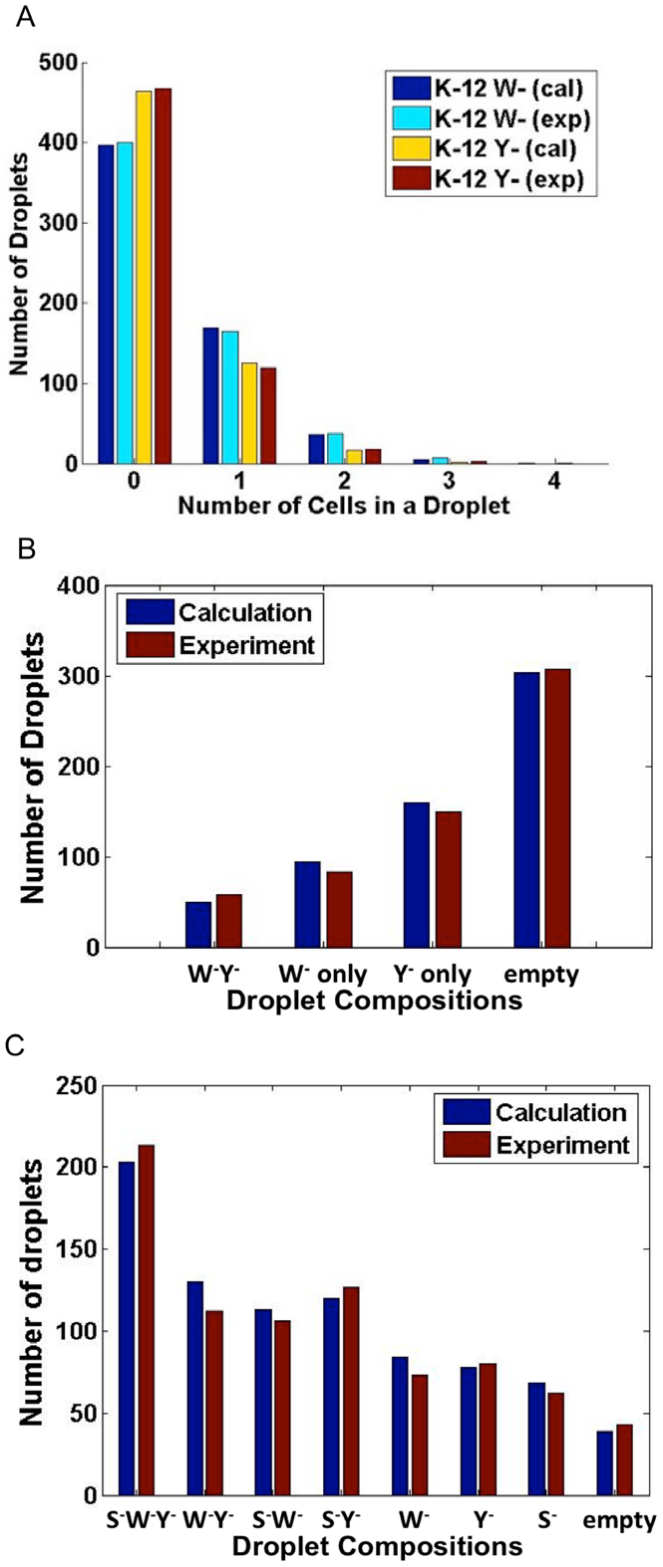
Comparisons between experiments and calculations for cell distribution in droplets. Calculations were based on the Poisson distribution. (A) Numbers of droplets carrying different numbers of cells. (B) Numbers of droplets carrying four different combinations of a two-strain system. (C) Numbers of droplets carrying eight different combinations of a three-strain system.

We hypothesized that compartmentalization of different microbial species in a community are independent events and for each species, the number of encapsulated cells in a droplet follows the Poisson distribution. For experimental validation of this hypothesis, a co-culture consisting of two differently labeled *E. coli* strains, named W^-^ and Y^-^, was injected into the device and the distribution of cells was examined with fluorescence microscopy. As shown in Fig. 2a, for each strain, the experimentally measured distribution of droplets carrying various numbers of cells agreed very well with calculated values using the Poisson distribution. The average number of cells in each droplet, which corresponded to the λ parameter in the Poisson distribution, was dependent upon the cell density in the seed culture injected into the device and the droplet volume determined by the device configuration.

As each species' distribution in droplets is expected to follow a simple Poisson distribution and different species are encapsulated independently, we could readily predict the distribution of the four combinations (i.e. W^-^ Y^-^, W^-^ only, Y^-^ only, and empty). Not surprisingly, there was a very good agreement between the calculated values and measured ones (Fig. 2b). This experimental validation was successfully extended to the distribution of a triplet system when a third strain, S^-^, was added (Fig. 2c). Therefore, the distribution of encapsulated subsets of a microbial community is highly predictable given the total cell concentration and composition of the community. Accordingly, for a given community composition, it is possible to determine the optimal cell density of the seed culture for a fixed device to achieve a desired droplet distribution.

### Co-cultivation of a symbiotic pair

**Figure 3:**
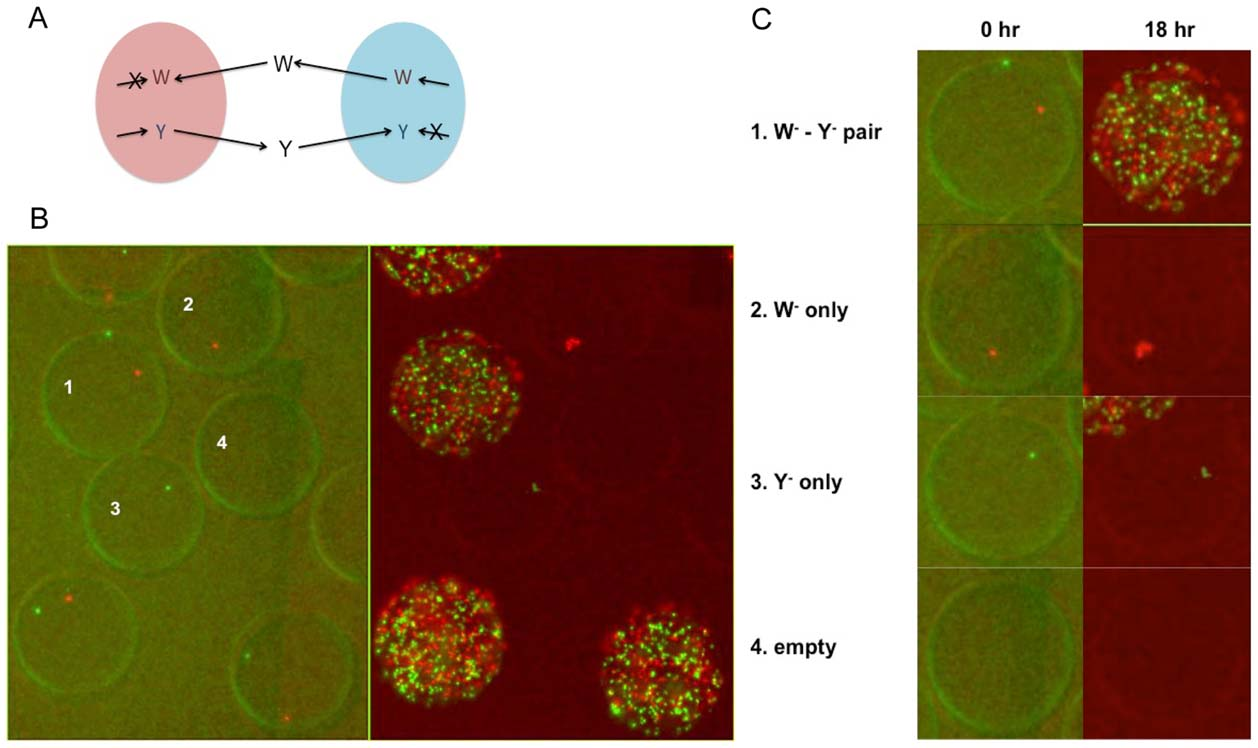
On-chip cultivation of a cross-feeding pair. (A) A synthetic symbiotic system consisting of two cross-feeding amino acid auxotrophs. (B) A section of the large cultivation chamber illustrating a number of droplets carrying four combinations of the two-strain system. *E. coli* strain Y^-^ is labeled with yellow fluorescence (EYFP) and W^-^ with red fluorescence (mCherry). Left – before cultivation, Right – Pictures after 18-hour cultivation. (C) Comparison of four individual droplets from Panel (B).

To mimic natural communities of interacting microbes, a synthetic symbiotic *E. coli* system consisting of a tryptophan auxotroph and a tyrosine auxotroph was constructed. Each auxotroph is unable to synthesize the corresponding amino acid and hence cannot survive in minimal media. However, when both auxotrophs are present, they can grow in the minimal medium by cross-feeding (Fig. 3a). To monitor the co-culture composition, each strain was labeled with a fluorescent protein and counted by microscopy.

On-chip co-cultivation of the tryptophan auxotroph (abbreviated to W^-^) and the tyrosine auxotroph (abbreviated to Y^-^) demonstrated that droplets could effectively compartmentalize co-cultures of microbes. Seed cultures of W^-^ and Y^-^ were diluted with the minimal medium and mixed properly such that the W^-^:Y^-^ ratio was 1∶1 and upon injection into the device, the average cell number per droplet was about 0.6. A total of 608 droplets were generated in this experiment. Of those, 317 were empty. 83 and 150 droplets had W^-^ only and Y^-^ only, respectively. 58 consisted of the W^-^ and Y^-^ pair.

After 18 hours of cultivation, only the cells in the droplets carrying both W^-^ and Y^-^ cells were growing well (Fig. 3b,c). We noted that some of the droplets carrying W^-^ or Y^-^ only were adjacent to droplets carrying the W^-^ and Y^-^ pair during cultivation, but cells in these droplets did not grow. This implied that the diffusion of amino acids did not occur across the droplet boundaries. In other words, the oil-water interface effectively blocked molecular diffusion between different droplets and therefore the droplets could generate highly parallelized and localized co-cultivation environments for detecting symbiotic relationships in a large and complex microbial community.

**Figure 4:**
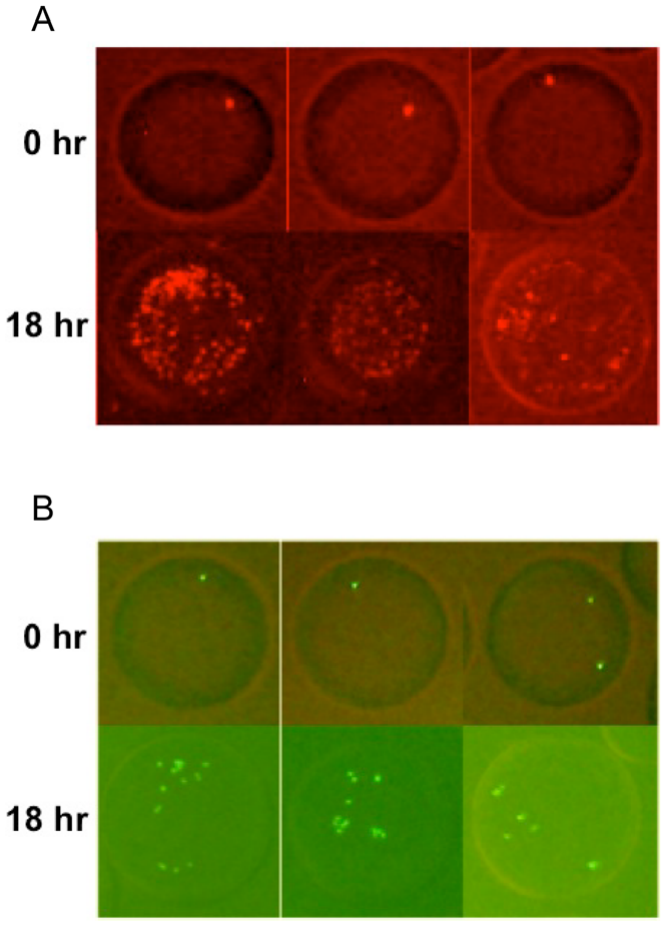
Comparison of a fast growing pair (K-12 W^-^ and Y^-^) and a slow growing pair (EcNR1 W^-^ and Y^-^) on the same device. (A) Three droplets carrying the pair of *E. coli* K-12 W^-^ expressing mCherry and Y^-^ (not labeled with fluorescence). Top panels – before cultivation. Bottom panels – after 18-hour cultivation. (B) Three droplets carrying the pair of *E. coli* EcNR1 W^-^ expressing GFP and K-12 Y^-^. Top panels – before cultivation. Bottom panels – after 18-hour cultivation.

On-chip droplet-based co-cultivation could further distinguish stronger symbiotic relationships from weaker ones among subsets of microbes. This could be demonstrated by examining two different strains of the tryptophan auxotroph when they were paired with the tyrosine auxotroph. We made use of a K-12 W^-^ strain and an EcNR W^-^ strain. Both grew with *E. coli* K-12 Y^-^ in the minimal medium, while the K-12 W^-^ and Y^-^ pair co-grew 50% faster than the EcNR W^-^ and Y^-^ pair in macro-scale tube cultures (growth rates: 0.189±0.011 hr^−1^ versus 0.126±0.004 hr^−1^). As shown in Fig. 4, when we injected a mixed culture of K-12 W^-^, EcNR W^-^, and Y^-^ with a ratio of 1∶1∶10 into the device, cells in droplets containing K-12 W^-^ and Y^-^ (Fig. 4a) grew significantly better than those in droplets containing EcNR W^-^ and Y^-^ (Fig. 4b).

### Co-cultivation of a triplet system

**Figure 5:**
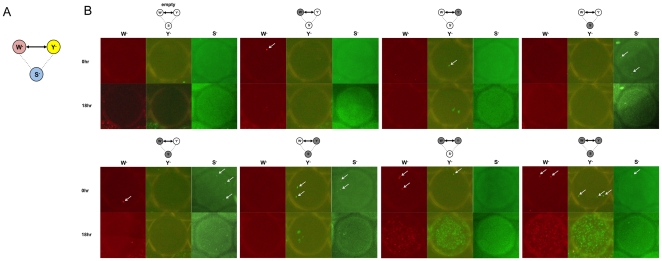
On-chip cultivation of a triplet system. (A) A synthetic system of three amino acid auxotrophs (W^-^, Y^-^, and S^-^). Only W^-^ and Y^-^ forms a symbiotic relationship. (B) Eight droplets illustrating all the combinations of the triplet system. Top panels are pictures taken before the cultivation and bottom panels are at 18 hours.

To mimic the complexity of natural communities more realistically, we introduced a third amino acid auxotroph, a serine auxotroph, into the system (Fig. 5a). The serine auxotroph (abbreviated to S^-^) was previously found to form no cross-feeding relationship with W^-^ or Y^-^ and hence could be considered as the background or noise in the community. We first examined the simplest case where the ratio of S^-^: W^-^: Y^-^ was 1∶1∶1. The average number of cells in each droplet was controlled to be three. For counting of cells and monitoring of growth, S^-^, W^-^ and Y^-^ were labeled with CFP, mCherry and EYFP, respectively. In this triplet system, eight different combinations were possible. A total of 816 droplets were generated and the number of droplets carrying each combination is shown in Table 1. As expected, among these droplets, only the cells in the droplets carrying the W^-^ and Y^-^ pair or the S^-^, W^-^ and Y^-^ triplet were able to grow, as shown in Table 1 and Fig. 5b. In particular, 112 droplets carried W^-^ and Y^-^ initially and 109 of them (97%) supported growth very well after 18 hours. This result showed that the droplet device was able to detect symbiotic relationships among subsets of members despite the presence of other microbes in the community.

**Table 1:** Number of droplets carrying various subsets of the triplet system.

(a)								
	empty	S^-^	W^-^	Y^-^	S^-^W^-^	S^-^Y^-^	W^-^Y^-^	S^-^W^-^Y^-^
growth	0	0	0	0	0	0	**109**	**210**
no growth	43	62	73	80	106	127	3	3
(b)								
	empty	S^-^	W^-^	Y^-^	S^-^W^-^	S^-^Y^-^	W^-^Y^-^	S^-^W^-^Y^-^
growth	0	0	0	0	0	0	**3**	**16**
no growth	40	153	138	2	515	6	1	2
(c)								
	empty	S^-^	W^-^	Y^-^	S^-^W^-^	S^-^Y^-^	W^-^Y^-^	S^-^W^-^Y^-^
growth	0	0	0	0	0	0	**1**	**5**
no growth	32	798	2	1	78	70	0	0

(a) S-: W-: Y- = 1∶1∶1 (b) S-: W-: Y- = 50∶50∶1 (c) S-: W-: Y- = 30∶1∶1.

In natural microbial communities, symbiotic partners might account for a small fraction of the total population. To mimic such conditions in nature, we further examined two other compositions of the *E. coli* triplet system. In one scenario, one partner of the symbiotic pair is a rare species; in the other, both partners are rare. In both cases, we tested the limit to which the device was able to detect the symbiosis between W^-^ and Y^-^ by generating droplets containing both and hence supporting co-growth of the pair. For the first case, we examined a mixed culture with the ratio of S^-^: W^-^: Y^-^ = 50∶50∶1, in which Y^-^ is a rare species accounting for about 1% of the total population. Based on simulation of cell distribution in droplets, we selected the average number of cells in each droplet to be five. To generate this average cell number per droplet, we estimated the density of the mixed culture under microscope using a Petroff-Hausser counting chamber and then diluted accordingly before injecting it into the device. Using a device with a 3 mm×10 mm incubation chamber, we generated a total of 880 droplets. As shown in Table 1b, because of the dominance of S^-^ and W^-^ in the population, the majority of the droplets contain either one or both of them, without the presence of the rare Y^-^. Nevertheless, four droplets turned out to contain the W^-^/Y^-^ pair, eighteen droplets encapsulated all three strains, and most of them (19 out of 22) showed well-sustained co-growth. It should be noted that for our synthetic system, the W^-^/Y^-^ pair can co-grow when S^-^ is present and hence the above two types of droplets (i.e. W^-^/Y^-^ and S^-^/W^-^/Y^-^) can both detect the existing symbiotic relationship. However, in natural microbial communities, due to negative interactions with other species[36], symbiotic partners may not be able to grow when other species are present. In this case, only the droplets that contain the symbiotic partners (e.g. W-/Y- in our model system) are desirable for supporting co-cultivation and detection of symbiotic relationships.

In the second case, we focused on the ratio of S^-^: W^-^: Y^-^ = 30∶1∶1, which exemplified symbiotic relationships existing between two rare species (here, each partner is about 3% of the total population). We chose to encapsulate an average of three cells in each droplet and generated a total of 977 droplets on a device with a 3 mm×10 mm chamber. In this scenario, a large fraction of the droplets contain S^-^, as detailed in Table 1c. Nevertheless, we managed to observe six droplets that encapsulated the W^-^/Y^-^ pair, including one containing only these two strains. All of these droplets supported the co-growth of W^-^/Y^-^ very well. The above results demonstrated that our device and co-cultivation method can effectively capture and amplify rare species in a microbial community and detect their symbiotic relationships with either abundant or other rare species.

## Discussion

In this work, we have demonstrated that microfluidically generated droplets can be effectively utilized to co-cultivate microbes and detect symbiotic relationships. Two features of our microfluidic device contributed to this effectiveness. First, due to its small dimensions and rapid operation, our droplets based device can achieve compartmentalization of microbial communities in a highly parallel and automated manner. Second, the interface between the aqueous phase in the droplet and the oil phase prevents molecular exchanges, and hence the cultivation environment in individual droplets is completely localized.

We used a synthetic *E. coli* symbiotic co-culture as a model system in this proof-of-concept study. Nevertheless, the platform we presented here for on-chip co-cultivation can be readily applied to a wide range of natural microbial communities. As shown in this work, the distribution of cells in droplets is highly predictable. Therefore, for a given microbial community with certain total density and composition, the design (e.g. droplet and chamber size) and operation (e.g. dilution ratio) of our microfluidic device can be optimized to co-cultivate and examine community members at different levels of abundance. For example, in this work, with up to 1,000 droplets, we could adjust the dilution ratio to detect the synthetic symbiotic interaction when one or both of the pair was very rare in the population. In addition, we showed that different extent of symbiosis could also be distinguished. Thus, our work suggests a promising new approach for cultivating microbes and for understanding microbial interactions. We can use this approach to cultivate and isolate various microbes of which the growth requires support (e.g. through signaling) from other species in the community. The cell cultures can then be further studied using a variety of characterization and analysis methods such as (meta)genome sequencing. Moreover, the co-growth data obtained from this co-cultivation approach will reveal positive relationships among community members, such as those between *Bifidobacterium adolescentis* and butyrate-producing anaerobes in the human gut[18], and negative ones. Elucidation of these microbes and their interactions might have important implications for many applications such as diagnostics and treatment of polymicrobial diseases.

This work represents an initial step towards the elucidation of microbe-microbe-environment interactions of complex communities based on microdroplet co-cultivation and characterization. To fulfill this long-term goal and to apply this approach to real microbial communities, we have identified two key tasks that require further efforts. First, automated droplet sorting and retrieval will enable us to distinguish droplets with well-developed mixed cultures from those with little growth and to obtain each of them individually. This can be achieved by coupling microscopy with flow control of droplets[37]. Second, when studying natural microbial communities, in which none of the cells is labelled, we need to characterize the retrieved droplet-mediated mixed cultures. Three genetic approaches with varying levels of details are suitable for this purpose. Terminal restriction fragment length polymorphism (T-RFLP) of 16 s rDNA is a simple method that can rapidly determine how many species are present in the cultivated communities[38]. Sequencing of 16 s rRNA will identify the cultured microbes at a species level. Finally, whole genome amplification followed by metagenome sequencing will potentially lead to comprehensive and detailed understanding of the genetic basis underlying the microbial interactions[39], [40]. We are currently investigating these microfluidic developments and off-chip characterization methods, which will facilitate the scale-up of the approach presented in this work for application to complex natural microbial communities.

## Materials and Methods

### Microfabrication

We used glass devices. Channels were fabricated using general photolithography processes. A glass wafer was prepared with Cr-Au deposition and AZ1518 spin coating. The pattern of the Cr mask was transferred to the AZ1518-coated glass wafer with the UV exposure of LI 300 for 30 seconds. After developing for 1 minute in MF319 developer, Au and Cr were etched for 2 minutes. The glass wafer was wet-etched by HF, until the depth of the channel reach 50 µm. The channel depth was measured periodically by depth profiler during the etching process. After dicing the individual devices, holes for inlets and outlets were electrochemically drilled in sodium hydroxide solution. To make the surface hydrophobic, a 2 µm-thick Parylene film was deposited on the channel and a cover slip. Afterwards, the channel and the cover slip were bonded with UV glue. Glass tubes were attached with UV glue at the holes as reservoirs of inlets and outlets, and syringe tips were fixed to the oil inlet and outlet reservoirs with epoxy to connect the device to vacuum source and oil reservoirs. Before using the devices for the cultivation, all devices were exposed to UV for at least one hour for sterilization. All devices were regenerated after each use. They were heated in a 540°C furnace for 2 hours, followed by cleaning in Piranha solution of H_2_O_2_:H_2_SO_4_ = 1∶2. Parylene coating, bonding with UV glue, attaching reservoirs and sterilization were repeated as described above.

### Construction of fluorescently-labeled synthetic symbiotic systems

Four *E. coli* strains were used in this work and all were amino acid auxotrophs constructed through the deletion of genes or operons encoding key enzymes in the amino acid biosynthesis pathways. K-12 W^-^, K-12 Y^-^, and K-12 S^-^ were constructed by deleting *trpE*, *tyrA*, and *serA*, respectively, in *E. coli* K-12. In addition, *tyrR* was deleted in K-12 W^-^ to increase the co-culture fitness when it is paired with K-12 Y^-^. All of the above gene deletions were carried out via P1 transduction with single-gene knock-out *E. coli* mutants from the Keio collection. After the transduction, the selection marker (*kan* gene) was deleted by transformation with the plasmid PCP20. The EcNR W^-^ strain was previously constructed by recombinogenic substitution of the *trpLEDCBA* operon with a selection marker (*cat* gene) in a derivative strain of *E. coli* MG1655 harboring a λ Red prophage. This tryptophan auxotroph strain grew at a slower rate when paired with K-12 Y^-^.

For labeling of different strains, four fluorescent proteins, mCherry, EYFP, CFP, and GFP, were utilized. mCherry and GFP were inserted into pET24b and EYFP into pET17b plasmids, respectively. The P_BAD_ promoter was inserted in front of the fluorescent gene, deleting the original T7 promoter and lac operator. Resulted plasmids were transformed into different strains as needed. CFP was integrated into the chromosome at the *galK* locus via P1 transduction with RP22[41] as the donor strain.

To prepare seed cultures for on-chip cultivation, cells expressing mCherry, GFP and EYFP were cultivated overnight in LB media containing 0.4% arabinose and cells labeled with CFP in LB media containing 0.2 mM IPTG. Then each strain was harvested and diluted 100 times in M9 minimal medium containing 0.4% arabinose and 0.2 mM IPTG. After the cell density of each seed culture was estimated using a Petroff-Hausser counting chamber and a fluorescence microscope, the diluted seed cultures were mixed to obtain the desired ratios.

### Encapsulation of microbes

A PFPE-PEG block copolymer surfactant[35] (RainDance Technologies) was dissolved in fluorinated oil HFE-7500 (3 M) at a final concentration of 2% wt/wt. The oil phase was supplied in a syringe connected to the device. After the device was connected to the vacuum source, 30 µl of diluted cell mixture was added into the aqueous-phase inlet reservoir. Using LabView interface, continuous vacuum was turned on, and the power of the vacuum was increased gradually to 150–300 mmHg. After enough droplets were generated, vacuum was turned off. All syringes and connections were removed from the device. 5 µl of mineral oil was added to each reservoir to prevent the evaporation of fluorinated oil.

### Cultivation and monitoring of microbes

As soon as droplet generation was completed, the device was examined by microscopy. Pictures at the beginning and the end of cultivation were taken by Olympus BX-51 and DP-71 with 20x objective lens. Exposure time and ISO were 0.25 sec/800, 0.8 sec/1600, and 0.2 sec/400 for mCherry, EYFP and CFP, respectively. For all pictures of CFP-expressing cells, autolevel and autocolor functions in Adobe Photoshop were applied to enhance the contrast. ImageJ was used to combine pictures from different channels. The device was placed in an incubator of 37°C for cultivation and pictures were taken as needed.
